# Distinct Swimming Behavioral Phenotypes Following Serotonin and Dopamine Transporter Modulation in the Adult Zebrafish Novel Tank Diving Test (NTT)

**DOI:** 10.3390/ph18121807

**Published:** 2025-11-27

**Authors:** Amaury Farías-Cea, Lisandra Pérez, Cristóbal Leal, Kerim Segura, Valentina Hernández, Caridad Atiés-Pérez, Luis Miguel Martínez, Martin Hödar-Salazar, Miguel Reyes-Parada, Ramón Sotomayor-Zárate, Francisca Rojas-Hidalgo, Marcela Julio-Pieper, Javier A. Bravo, Dasiel O. Borroto-Escuela, Patricio Iturriaga-Vásquez

**Affiliations:** 1 Laboratorio de Farmacología Molecular y Química Medicinal, Departamento de Ciencias Química y Recursos Naturales, Facultad de Ingeniería y Ciencias, Universidad de La Frontera, Temuco 4811230, Chile; lisandraperez0118@gmail.com (L.P.); c.leal13@ufromail.cl (C.L.); k.segura01@ufromail.cl (K.S.); v.hernandez06@ufromail.cl (V.H.); atiescary93@gmail.com (C.A.-P.); miguetzd@gmail.com (L.M.M.); mhodar@uct.cl (M.H.-S.); 2 Laboratorio de Bioquímica y Farmacología Molecular, Escuela de Ciencias, Facultad de Ciencias de la Vida, Universidad Viña del Mar, Viña del Mar 2572007, Chile; 3 Programa de Doctorado en Ciencias de Recursos Naturales, Universidad de la Frontera, Temuco 4811230, Chile; 4 Programa de Doctorado en Ciencias Mención Biología Celular y Molecular Aplicada, Universidad de La Frontera, Temuco 4811230, Chile; 5 Departamento de Procesos Diagnósticos y Evaluación, Facultad de Ciencias de la Salud, Universidad Católica de Temuco, Temuco 4813302, Chile; 6 Centro de Investigación Biomédica y Aplicada (CIBAP), Escuela de Medicina, Facultad de Ciencias Médicas, Universidad de Santiago de Chile, Estación Central 9170022, Chile; miguel.reyes@usach.cl; 7 Facultad de Ciencias de la Salud, Universidad Autónoma de Chile, Santiago 7500912, Chile; 8 Centro de Neurobiología y Fisiopatología Integrativa (CENFI), Instituto de Fisiología, Facultad de Ciencias, Universidad de Valparaíso, Valparaíso 2360102, Chile; ramon.sotomayor@uv.cl; 9 Grupo NeuroGastroBioquímica, Instituto de Química, Facultad de Ciencias, Pontificia Universidad Católica de Valparaíso, Valparaíso 2340000, Chile; francisca.rojas@pucv.cl (F.R.-H.); marcela.julio@pucv.cl (M.J.-P.); javier.bravo@pucv.cl (J.A.B.); 10 Receptomics and Brain Disorders Lab, IBIMA Plataforma BIONAND, Department of Human Physiology, Physical Education and Sport, Faculty of Medicine, University of Malaga, 29071 Málaga, Spain; dasiel@uma.es

**Keywords:** serotonin transporter, dopamine transporter, fluoxetine and methylphenidate, Novel Tank Test, bottom-dwelling

## Abstract

**Background/Objective:** Serotonin and dopamine are key neurotransmitters involved in regulating mood, anxiety, and locomotor activity. Specific transporters mediate their reuptake, SERT and DAT, making them targets for drugs such as Fluoxetine and Methylphenidate. Zebrafish (*Danio rerio*), due to their genetic and neurochemical similarity to humans, serve as a valuable model for studying the behavioral effects of these drugs. This study aimed to compare the behavioral phenotypes induced by SERT and DAT blockers in adult zebrafish using the Novel Tank Diving Test (NTT), thereby generating a swimming profile for drugs acting on these monoamine transporters that can be utilized in drug discovery and behavior. **Methods**: Adult zebrafish were administered Fluoxetine or Methylphenidate and subjected to the NTT. Behavioral endpoints measured included bottom-dwelling time (anxiety-like behavior), swimming velocity (locomotor activity), and transitions to the upper zone (exploratory behavior). **Results**: Fluoxetine treatment significantly reduced bottom-dwelling behavior, increased transitions to the upper zone, and decreased erratic swimming, indicating reduced anxiety and enhanced exploration. In contrast, Methylphenidate administration led to prolonged bottom-dwelling and reduced exploration, suggesting increased anxiety-like behavior and decreased exploration. These findings highlight distinct behavioral profiles resulting from selective modulation of serotonergic and dopaminergic pathways. **Conclusions**: The study demonstrates that SERT and DAT blockades produce divergent behavioral effects in adult zebrafish, with Fluoxetine exhibiting anxiolytic and exploratory-promoting actions. At the same time, Methylphenidate induces anxiety-like and less exploratory behaviors. These results underscore the utility of zebrafish as a valuable translational model for neuropharmacological research and drug discovery, providing insights into the differential impact of serotonergic and dopaminergic modulation on behavior.

## 1. Introduction

Neurotransmitters are chemical substances released from synaptic neurons that exert specific cellular effects [1]. There is a wide range of neurotransmitters, which can be divided into three categories: aminoacidic neurotransmitters, peptide neurotransmitters, and neurotransmitters derived from aromatic amino acids. Dopamine (DA), norepinephrine (NE), and serotonin (5-HT) are monoamine neurotransmitters that fall into the last category [2]. DA is involved in functions such as movement, learning, and motivation. Also, it plays a key role in the neurobiology of addiction [3]. NE regulates stress responses and arousal [4,5], while 5-HT regulates mood, arousal, appetite, impulsiveness, aggression, sleep, and cognitive functions [6,7]. To maintain the balance of monoamines released from presynaptic neurons into the synaptic cleft, a family of transporters regulates the reuptake of such neurotransmitters [8,9]. The SLC6A gene family encodes these transporters, which are named according to their substrate transport: SERT for the serotonin transporter, DAT for the dopamine transporter, and NET for the norepinephrine transporter [9]. These transporters are highly expressed in the brain and, due to their function, are the targets of drugs that cause profound behavioral effects and exhibit therapeutic usefulness in the treatment of conditions such as depression, obsessive–compulsive disorder, and attention deficit hyperactivity disorder (ADHD), among other pathologies [10,11].

Fluoxetine was synthesized in 1971 and described in the scientific literature in 1974 by Eli Lilly Pharmaceuticals (LY110140) [12]. It was the first compound proposed as an inhibitor of 5-HT reuptake [13]. It took around 17 years for Fluoxetine hydrochloride (PROZAC) to be fully developed and approved by the US FDA for the treatment of depression in 1987 [14], giving rise to a new class of antidepressant drugs commonly known as selective serotonin reuptake inhibitors (SSRIs). *Fortune* magazine named it the “Pharmaceutical Product of the Century” in 1999, with more than 40 million patients treated worldwide [14]. Fluoxetine is a well-established antidepressant and effective for the treatment of anxiety symptoms in children, adolescents, and adults, with most studies reporting good tolerability and only mild, transient side effects such as headache or gastrointestinal discomfort [15,16,17]. However, in animal models and in some adolescent populations, chronic Fluoxetine may paradoxically increase anxiety in a dose-dependent manner, suggesting the need for careful monitoring in younger patients [18]. In adults with major depression, Fluoxetine reduces both depressive and anxiety symptoms, but discontinuation of treatment due to side effects is more common than with placebo [19].

Methylphenidate was first synthesized in 1944 [20], marketed by Ciba-Geigy Pharmaceutical Company as “Ritalin” in 1954, and approved for sale in the United States in 1955 [21,22]. Methylphenidate has been approved by the FDA for the treatment of ADHD in children and adolescents, as well as for narcolepsy in adults [23,24]. It is also used to treat refractory depression in the geriatric population with Alzheimer’s disease, improving cognitive function, among other benefits [25,26,27,28]. The primary targets of Methylphenidate are DAT (Ki = 84 nM) and NET (Ki = 514 nM), where it inhibits the reuptake of DA and NE into their presynaptic neurons in the CNS [29,30,31]. Methylphenidate, commonly prescribed for ADHD, shows mixed effects on anxiety. In children and adults with ADHD, prolonged Methylphenidate treatment often reduces anxiety, especially in those with pre-existing anxiety symptoms [32,33,34]. However, acute or single-dose administration may provoke or increase anxiety in some pediatric patients, potentially contributing to early treatment discontinuation [35]. In healthy adults, Methylphenidate can increase subjective anxiety, particularly under stress or cognitive load [36,37]. Animal studies indicate that Methylphenidate may have anxiolytic effects at specific doses, but chronic use in young subjects could increase anxiety later in life [38,39].

Diverse animal models are used to study the behavioral properties of drugs, with murine models being the most common. However, over the last decade, zebrafish have emerged as a solid alternative to murine models, in principle due to the high similarity between their genome and the human genome [40]. At least 70% of human genes have one ortholog in zebrafish, and 82% of the genes related to human diseases, listed in the Online Mendelian Inheritance in Man database, have at least one corresponding zebrafish ortholog [41]. Additionally, its active behavior, ease of acclimation to new environments, ease of manipulation, low maintenance costs, and rapid reproductive cycle make the zebrafish a valuable model for investigating human pathologies [42,43]. Importantly, beyond their genetic homology, zebrafish exhibit a wide range of complex behaviors, including social interactions, learning, memory, stress responses, and anxiety-like behaviors. This behavioral repertoire enables researchers to utilize zebrafish for detailed behavioral assessments, providing valuable insights into neurotoxicity, neurodevelopmental disorders, and other neurological or psychiatric conditions [44,45]. In addition, zebrafish are an efficient model for drug discovery and screening because they are susceptible to drugs from various classes of drugs related to neuropharmacology, such as Amphetamine, Nicotine, Methylphenidate, Cytisine derivatives, Noribogaine, Atropine, Scopolamine, and Ethanol [46,47,48,49,50,51,52]. Behavioral analysis in zebrafish is commonly performed using standardized tests, with the Novel Tank Diving Test (NTT) among the most widely used. The NTT enables the assessment of anxiety-like and exploratory behaviors, making it a powerful tool for evaluating the effects of pharmacological agents and environmental factors on zebrafish behavior [53,54].

Despite differences in brain structure between zebrafish and humans, many proteins, such as those in the SLC6 family, are relatively conserved, suggesting that molecular mechanisms will operate similarly in both species. However, in the case of SERT, there are two distinct protein transporters, SERTa and SERTb [55], but their functions and distributions are not fully elucidated. In terms of brain structure, although different, there is evidence that similar neural circuits for executive function exist in zebrafish and rats [56]. For example, the teleost pallium contains homologous structures to the mammalian amygdala, which is hyperactivated in clinical anxiety, social anxiety disorder, and drug abuse, and the zebrafish pallium shows an increase in FOS protein (neuronal activation) during drug-seeking behavior and Amphetamine administration. Consequently, the pallium is postulated to be a homolog of the mammalian amygdala, with evolutionarily conserved functions in modulating key behaviors [57].

Zebrafish models allow investigation of neurobehavioral toxicity, risk-taking, sociability, high-throughput behavioral screening, and the molecular mechanisms underlying drug action, which are often conserved across vertebrates. Furthermore, the increasing prevalence of these medications, their potential for misuse, and the risk of adverse effects, including anxiety, neurodevelopmental changes, and even transgenerational impacts, underscore the need for comprehensive behavioral characterization. Such research not only advances our understanding of drug safety and efficacy but also informs clinical practice, drug development, and public health strategies [43,58,59,60,61].

Therefore, the present study aims to characterize the behavioral effects of Fluoxetine and Methylphenidate in zebrafish using the NTT, to provide insights into their impact on anxiety-like and exploratory behaviors, and to contribute to the translational relevance of zebrafish as a model for neuropsychiatric drug research.

## 2. Results

### 2.1. Bottom-Dwelling Time

**Figure 1:**
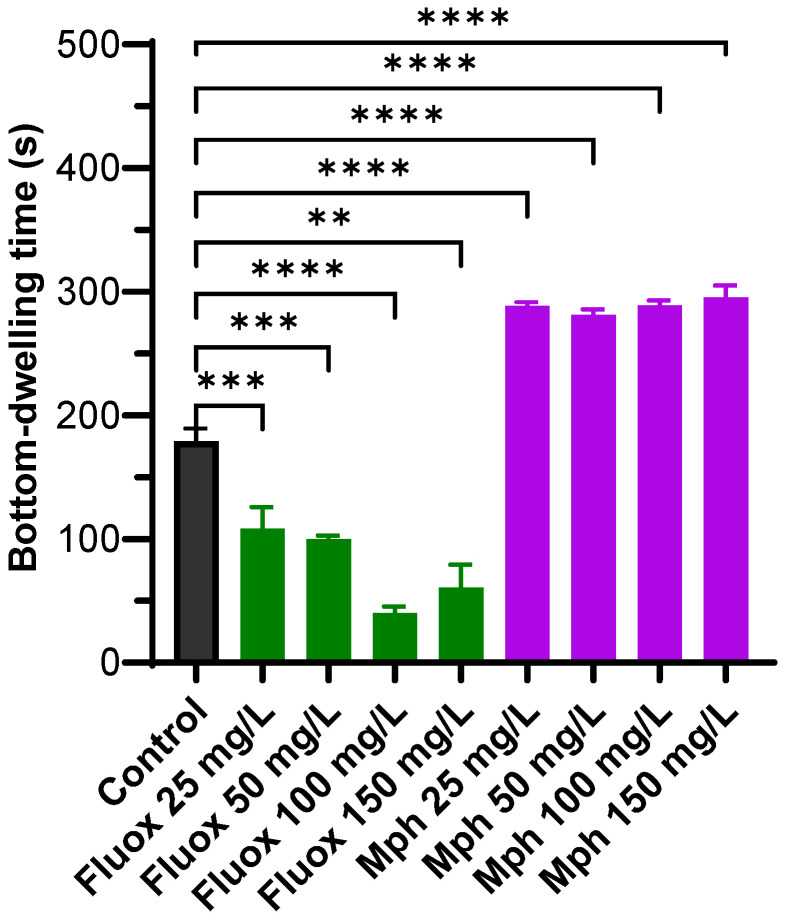
Bottom-dwelling time for control (black), Fluoxetine (Fluox) (green), and Methylphenidate (Mph) (purple) at 25, 50, 100, and 150 mg/L. Fishes were exposed to the drugs for 3 min and kept for another 5 min in a holding tank (without drug) before starting the swimming behavior protocol in the NTT for 5 min. The number of animals was *n* = 10 for each experiment. Multiple comparisons against the control by the Brown–Forsythe and Welch ANOVA. ** *p* ≤ 0.01, *** *p* ≤ 0.001 and **** *p* ≤ 0.0001.

Adult fish were exposed to Fluoxetine and Methylphenidate (*n* = 10 per compound/dose) (Figure 1), and their swimming behavior was measured using the NTT protocol for 5 min. All drugs were used at four different concentrations: 25, 50, 100, and 150 mg/L. These concentrations were selected based on our experience with administering drugs to adult zebrafish and are consistent with other published data [49,62,63,64].

The results show that, compared to the control (179.0 ± 34.9 s), the SERT blocker Fluoxetine significantly reduces the time spent at the bottom of the fish tank at all concentrations used: 108.7 ± 28.3 s for 25 mg/L (*p* = 0.0005), 99.88 ± 11.0 s for 50 mg/L (*p* = 0.0002), 40.2 ± 22.4 s for 100 mg/L (*p* < 0.0001), and 60.7 ± 25.3 s for 150 mg/L (*p* = 0.0013. Additionally, the well-known DAT blocker Methylphenidate significantly increased bottom-dwelling time compared to the control at all concentrations assayed (288.4 ± 11.3 s at 25 mg/L (*p* < 0.0001), 281.4 ± 12.0 s at 50 mg/L (*p* < 0.0001), 288.8 ± 12.7 s at 100 mg/L (*p* < 0.0001), and 295.4 ± 20.2 s at 150 mg/L (*p* < 0.0001)). Overall, the bottom-dwelling behavior shows an increase in time spent at the bottom of the fish tank for Methylphenidate, whereas for Fluoxetine, time spent at the bottom decreased at all concentrations tested.

### 2.2. Average Velocity

**Figure 2:**
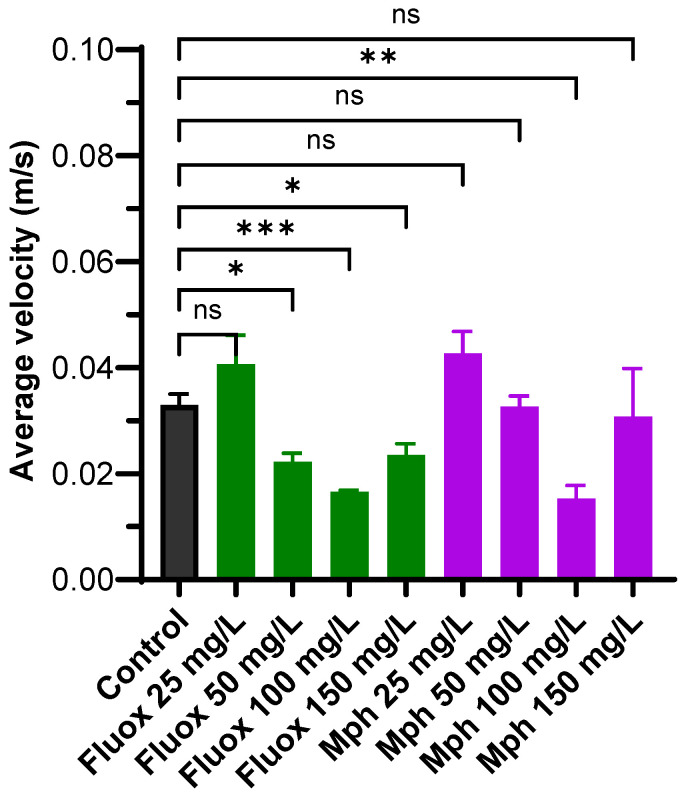
Average velocity for control (black), Fluox (green), and Mph (purple) at 25, 50, 100, and 150 mg/L in the Novel Tank Diving Test. The number of animals was *n* = 10 for each experiment. Multiple comparisons against the control by the Brown–Forsythe and Welch ANOVA. ns: Not significant, * *p* ≤ 0.1, ** *p* ≤ 0.01, *** *p* ≤ 0.001.

The average velocity for the control group was 0.032 ± 0.006 m/s. For Fluoxetine, our results show that, except for 25 mg/L (0.040 ± 0.005 m/s, *p* = 0.1742), the average velocity decreased significantly compared with the control: 0.022 ± 0.004 m/s at 50 mg/L (*p* = 0.0119); 0.017 ± 0.001 m/s at 100 mg/L (*p* = 0.0008), and 0.023 ± 0.006 m/s at 150 mg/L (*p* = 0.0482). For Methylphenidate, the average velocity was: 0.042 ± 0.009 at 25 mg/L (*p* = 0.3450), 0.032 ± 0.005 m/s at 50 mg/L (*p* > 0.9999), 0.015 ± 0.006 m/s at 100 mg/L (*p* = 0.0015), and 0.03 ± 0.009 m/s at 150 mg/L (*p* = 0.09993). These results show that Methylphenidate was not significantly different from the control; however, at 100 mg/L, it exerted a significant decrease in velocity (0.015 ± 0.007 m/s) (Figure 2).

### 2.3. Transitions to the Upper Half

**Figure 3:**
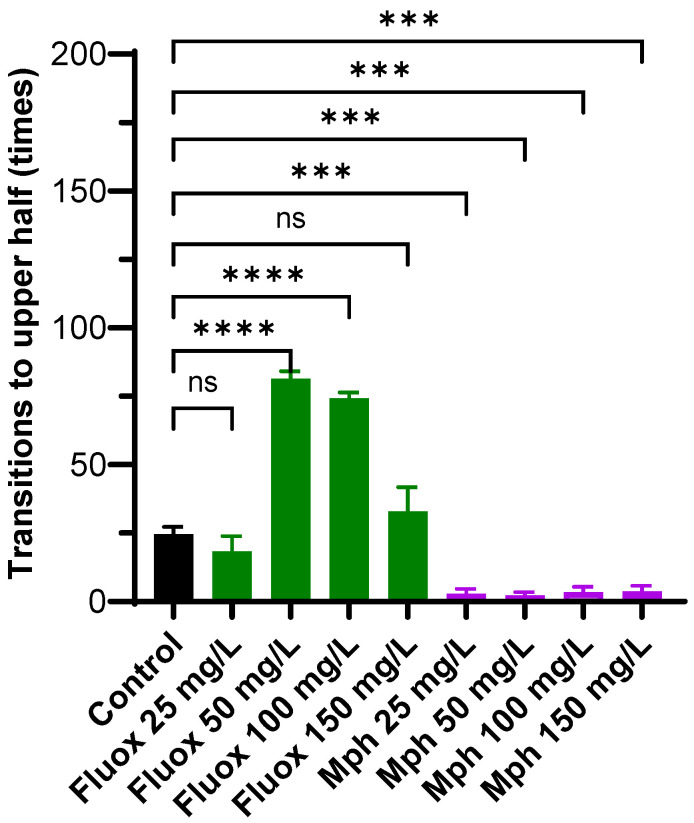
Transitions to the upper half for control (black), Fluox (green), and Mph (purple) at 25, 50, 100, and 150 mg/L in the Novel Tank Diving Test. The number of animals was *n* = 10 for each experiment. Multiple comparisons against the control by the Brown–Forsythe and Welch ANOVA. ns: Not significant, *** *p* ≤ 0.001 and **** *p* ≤ 0.0001.

The number of times that the fish moved from the bottom to the top was measured (Figure 3). For the control condition, the number of transitions was 24.7 ± 7.4 times. For Fluoxetine, a significantly higher number of transitions compared to the control was observed 81.5 ± 11.4 times at 50 mg/L (*p* < 0.0001) and 74.3 ± 7.2 times at 100 mg/L (*p* < 0.0001), whilst the transition to the upper at 25 mg/L (18.4 ± 5.4 times, (*p* = 0.5336) and 150 mg/L (32.8 ± 9.7 times, *p* = 0.9498) were not different compared with the control. In contrast, the number of crossings was significantly decreased for Methylphenidate at all concentrations tested (2.8 ± 1.9 times at 25 mg/L (*p* = 0.0001), 2.3 ± 1.7 times at 50 mg/L (*p* = 0.0001), 3.4 ± 2.8 times at 100 mg/L (*p* = 0.0001), and 3.8 ± 2.5 times at 150 mg/L (*p* = 0.0005)).

### 2.4. Erratic Movements

**Figure 4:**
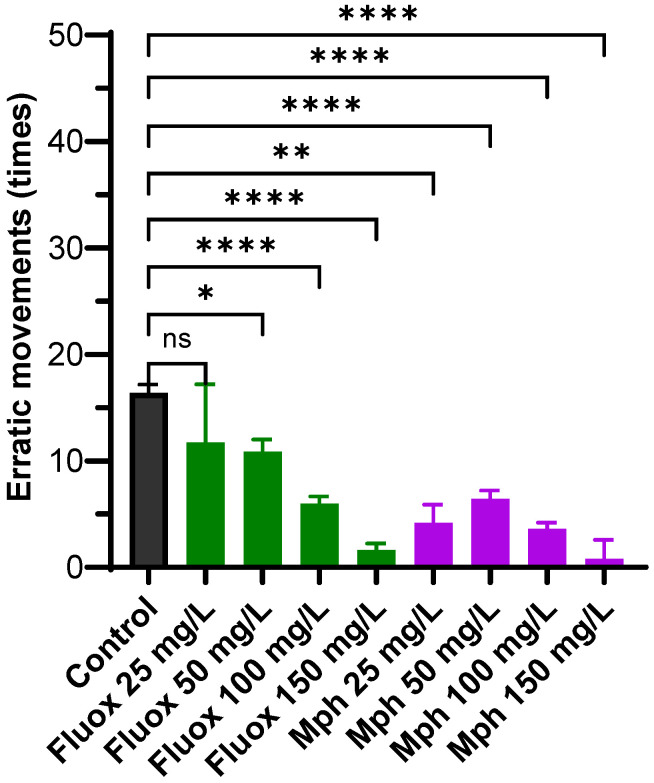
Erratic movements for control (black), Fluox (green), and Mph (purple) at 25, 50, 100, and 150 mg/L, in the Novel Tank Diving Test. The number of animals was *n* = 10 for each experiment. Multiple comparisons against the control by the Brown–Forsythe and Welch ANOVA. ns: Not significant, * *p* ≤ 0.1, ** *p* ≤ 0.01, and **** *p* ≤ 0.0001.

Analysis of the number of unexpected movements of the fish in the novel tank shows that both compounds tested induced fewer erratic movements than the control (16.5 ± 2.6 times). For Fluoxetine, the erratic movements were 8.8 ± 6.2 times for 25 mg/L (*p* = 0.6290), 11.0 ± 3.2 times for 50 mg/L (*p* = 0.0124), 6.0 ± 1.8 times for 100 mg/L (*p* < 0.0001), and 1.6 ± 0.9 times for 150 mg/L (*p* < 0.0001), respectively. For Methylphenidate, this value decreased to 4.2 ± 2.1 times at 25 mg/L (*p* = 0.0040), 6.2 ± 2.2 times at 50 mg/L (*p* < 0.0001), 3.7 ± 1.6 times at 100 mg/L (*p* < 0.0001), and 0.8 ± 0.7 times at 150 mg/L (*p* < 0.0001). Our results show that Fluoxetine and Methylphenidate decreased erratic movements; for Fluoxetine, higher concentrations were more significant, and for Methylphenidate, the decrease was displayed at all concentrations assayed (Figure 4).

## 3. Discussion

In this study, we investigated swimming behavior in adult zebrafish (*Danio rerio*) using the NTT, a well-established behavioral test for assessing anxiolytic-like responses and exploratory activity. In this assay, time spent in the lower versus upper regions of the tank is indicative of anxiety-related states: increased bottom-dwelling is associated with anxiogenic or stress-like behavior [65], whereas increased time in the upper zone reflects anxiolytic-like effects. Behavioral endpoints included bottom-dwelling time, average swimming velocity (as a measure of locomotor activity), number of transitions to the upper tank zone (exploratory behavior), and incidence of erratic movements. These metrics were evaluated following an acute exposure to Fluoxetine, a selective serotonin reuptake inhibitor (SERT blocker), and Methylphenidate, a dopamine reuptake inhibitor (DAT blocker), to compare the behavioral effects of pharmacological modulation of monoaminergic signaling. We also performed conventional PCR experiments to confirm the presence of DAT, SERTa, and SERTb genes, which allowed us to associate the actions of Fluoxetine and Methylphenidate with their respective transporters and their modulation of neurotransmitters (Supplementary Information).

In the NTT, increased bottom-dwelling is widely interpreted as an anxiety-like response, while reduced bottom-dwelling (i.e., more time spent near the surface) reflects reduced anxiety [66,67,68]. This behavior is validated by the fact that anxiolytic drugs (e.g., Fluoxetine, diazepam) decrease bottom-dwelling, while anxiogenic (anxiety-inducing) agents increase it [69,70]. Our results demonstrate pronounced differences in the bottom-dwelling between treatments. Fluoxetine significantly reduced bottom-dwelling time. Our results agree with studies using chronic administration in adult zebrafish [57]. In contrast, Methylphenidate increased bottom-dwelling time by approximately 40% across all tested concentrations relative to controls. The significant reduction in bottom-dwelling time by Fluoxetine at all tested concentrations indicates a strong anxiolytic effect, consistent with its effects in previous studies [66,67]. This effect is robust and dose-dependent, mirroring findings in both zebrafish and mammalian models [69,70].

The increase in bottom-dwelling time induced by Methylphenidate suggests an anxiogenic effect, suggesting that Methylphenidate may heighten anxiety-like behavior in zebrafish under these conditions. This aligns with reports showing that psychostimulants can provoke anxiety-like responses, especially at specific doses or in acute settings [66,67].

Average swimming velocity, which is usually used as an indicator of locomotor activity, decreased after either Fluoxetine or Methylphenidate. A decreased swimming velocity is often interpreted as a sign of sedation, motor impairment, or neurotoxicity, rather than a direct reflection of anxiety levels [71,72,73,74]. Thus, the effect elicited by Fluoxetine could be linked to its action upon serotonergic signaling, which could disrupt normal motor function, inducing sedative-like effects [75]. Methylphenidate decreases velocity at high concentrations, suggesting possible neurotoxic or sedative effects, despite its anxiogenic profile in bottom-dwelling behavior. This may reflect overstimulation or disruption of dopaminergic and noradrenergic pathways, leading to reduced motor output [72]. Reduced swimming velocity from both drugs could reflect sedative or neurotoxic effects on the nervous system, not just changes in anxiety. This highlights the importance of distinguishing between anxiety-related and general motor effects in this behavioral model. Furthermore, the opposing effects of SERT and DAT blockers on bottom-dwelling time and locomotor activity support the notion that distinct behavioral profiles may be attributed to specific mechanisms of action.

The number of transitions to the upper zone, reflecting exploratory activity, increased markedly with Fluoxetine treatment. In contrast, Methylphenidate treatment led to a consistent decrease in upper zone transitions across all concentrations tested. These findings suggest that only Fluoxetine enhances exploratory behavior, whereas Methylphenidate produces the opposite effect. These changes in upper zone transitions are widely interpreted as indicators of anxiety-like states and altered stress responsiveness in zebrafish. The dose-dependent increase in upper zone transitions induced by Fluoxetine is consistent with previous reports, which have associated enhanced serotonergic signaling with reduced anxiety and increased willingness to explore novel environments [75,76]. Notably, this effect was more pronounced at intermediate concentrations, with higher or lower doses showing less effect or even sedative outcomes [77]. On the other hand, the decrease in the explorative transitions elicited by Methylphenidate suggests an anxiogenic or suppressive effect on motivation and exploration, possibly due to overstimulation or disruption of dopaminergic/noradrenergic pathways [72].

Both compounds dose-dependently reduce erratic movements, although Methylphenidate appears to be more potent across all tested concentrations. Reductions in erratic movements in zebrafish are widely interpreted as decreases in anxiety-like behavior and improvements in motor control. The anxiolytic-like effects of Fluoxetine, which are likely due to its enhancement of serotonergic signaling, are consistent with decreased anxiety-like behaviors, lower cortisol levels, and altered anxiety-related gene expression reported in other studies [70,71,72,73,74]. On the other hand, Methylphenidate reduces erratic movements, likely via dopaminergic and noradrenergic pathways [78]. These findings highlight distinct pathways for modulating anxiety and motor control in aquatic models [70,78].

In summary, our results indicate that an acute administration of Fluoxetine exhibits a behavioral profile similar to that of anxiolytic substances, such as benzodiazepines, buspirone, SSRIs, synthetic chalcones, ibuprofen derivatives, paracetamol, nicotine, and numerous plant-derived compounds. [47,62,79,80,81,82,83,84]. On the other hand, Methylphenidate induced mixed anxiogenic/anxiolytic effects (depending on the behavior considered). In summary, changes in bottom residence time and transitions to the upper zone are the main behavioral differences between these two substances, suggesting that drugs that act as SERT or DAT blockers produce distinct swimming profiles that could be used to discover new drugs targeting SERT and DAT. Furthermore, our results support the notion that zebrafish is a versatile model for screening both established and novel anxiolytic agents.

## 4. Materials and Methods

### 4.1. Animals

A total of 100 adult, zebrafish wild-type, 6–8 months old, male–female ratio 1:1, were used to perform the behavioral assays. Fish were obtained from a local commercial distributor (Natural Fish, Peñaflor, Chile) and housed in a fish tank system with filtered system water, maintained at 26–28 °C, and subjected to a constant 14:10 h light/dark cycle. Experiments were performed after at least one week of acclimatization to our lab conditions, during which fish were fed twice daily with the “Vipan food base” from Sera and observed for any unusual behavior. All the experiments were performed during light hours, and after every trial, the fish were euthanized using benzocaine and ice-cold water. Animal care and experimentation were conducted in accordance with national regulations.

### 4.2. Drugs and Treatment

Fluoxetine hydrochloride (F132, Sigma-Aldrich, Saint Louis, MO, USA) and Methylphenidate hydrochloride (M2892, Sigma-Aldrich, Saint Louis, MO, USA) used in this experiment were provided by Dr. Ramón Sotomayor-Zárate and Dr. Miguel Reyes-Parada, respectively. Drugs were dissolved in system water and administered to fish by immersion in a beaker containing the drug solution at the required concentration for each experiment. Adult fish were exposed to Fluoxetine and Methylphenidate for 3 min, then placed in a holding tank (without the drugs) for 5 min before initiating the swimming behavior protocol in the NTT for 5 min. All drugs were used at four different concentrations: 25, 50, 100, and 150 mg/L. These concentrations were selected based on our experience with administering drugs to adult zebrafish and are consistent with other published data [47,58,59,60].

### 4.3. Novel Tank Diving Test

The novel tank diving test (NTT) used was an acrylic trapezoid (14.5 cm height × 27 cm top × 22 cm bottom × 16 cm diagonal × 5 cm width), filled with 1.6 L of water. The room temperature was adjusted to 28 °C. For the test, zebrafish were individually moved from their housing tank to a holding tank (filled with water) to acclimate for five minutes. Once the acclimatization time was over, the fish were transferred to another tank with the drug dissolved (*n* = 10 per dose at 25, 50, 100, and 150 mg/L for Fluox and Mph) or regular water in the case of the control group, for a 3 min period. After the drug exposition, the fish was kept in a second holding tank without drugs for five minutes and finally moved to the test tank (NTT) for five minutes, where the swimming was recorded with a USB camera. A white acrylic box covered the test tank to avoid lighting issues, and a white acrylic screen backlit the tank to provide good contrast for proper fish detection. Swimming behaviors were analyzed from recorded videos using Noldus Ethovision XT 14.0 software (Wageningen, The Netherlands). The video analysis focused on anxiety-related parameters (time spent in the bottom), motor-related parameters (average velocity, calculated as the total distance traveled over the time recording), and exploratory activity (number of transitions to the upper half) [47,58,63].

### 4.4. Statistical Analysis

Data were analyzed using the Brown–Forsythe and Welch ANOVA tests, followed by the Dunnett T3 multiple comparisons test. The statistical analysis and data visualization were performed using GraphPad Prism 10.4.1 (Boston, MA, USA) for Windows, which does not assume equal standard deviations (or homogeneity of variance). The data are presented as the mean ± SEM, with *p* values ≤0.1, ≤0.01, ≤0.001, and ≤0.0001 regarded as statistically significant.

## 5. Conclusions

Our findings demonstrate that Fluoxetine and Methylphenidate induce markedly distinct behavioral phenotypes in zebrafish, as assessed using the NTT. The behavioral profile observed following Fluoxetine administration, characterized by a significant reduction in bottom-dwelling time, an increase in the number of transitions to the upper half of the tank, and a reduction in erratic swimming, aligns with the established anxiolytic properties of selective serotonin reuptake inhibitors (SSRIs) in humans and is consistent with previously reported outcomes in zebrafish models.

In contrast, the behavioral effects elicited by Methylphenidate are less straightforward. Methylphenidate exposure was associated with increased bottom-dwelling, along with an unanticipated reduction in average swimming velocity. Interestingly, Methylphenidate also produced a similar decrease in erratic movements as Fluoxetine, despite the divergent effects on exploration and bottom-dwelling behaviors. These findings may reflect the more complex and context-dependent role of Methylphenidate in modulating anxiety-related processes.

Some limitations, such as the number of animals and the exclusive focus on acute exposure, could be addressed to improve the study. Future studies should consider larger cohorts and investigate chronic administration to better understand the long-term behavioral and neurochemical consequences of these compounds. Furthermore, exploring the underlying molecular mechanisms and extending the analysis to other behavioral paradigms could provide additional insights into the complex actions of fluoxetine and methylphenidate.

In conclusion, Fluoxetine promotes an anxiolytic-like phenotype in zebrafish, evidenced by increased exploration and time spent in the upper tank zone. In contrast, Methylphenidate induces behavioral responses indicative of reduced exploratory activity and a potentially anxiogenic-like profile. These distinct behavioral signatures highlight the differential roles of serotonergic and dopaminergic pathways in regulating zebrafish swimming behavior, underscoring the utility of the NTT as a platform for pharmacological screening and neurobehavioral profiling of monoaminergic compounds.

## Data Availability

The original contributions presented in this study are included in the article/Supplementary Material. Further inquiries can be directed to the corresponding authors.

## Supplementary Materials

The following supporting information can be downloaded at: https://www.mdpi.com/article/10.3390/ph18121807/s1, Figure S1: PCR gel of GAPDH, DAT, SERTa, SERTb, and ACTb1; Table S1: Sequence of primers used in the RT-PCR analysis.
