# Supplementary material for: Distinct Swimming Behavioral Phenotypes Following Serotonin and Dopamine Transporter Modulation in the Adult Zebrafish Novel Tank Diving Test (NTT)
Source: Pharmaceuticals (Basel). 2025 Nov 27;18(12):1807. doi: 10.3390/ph18121807 (PMC12735686; doi:10.3390/ph18121807)
Supplement: Supplementary file 1 [file pharmaceuticals-18-01807-s001.zip › pharmaceuticals-3976555-supplementary.pdf]

Reverse Transcription Polymerase Chain Reaction (RT-PCR)

We examined the mRNA expression levels of the brain's Dopamine Transporter (DAT) and Serotonin Transporter (SERT). Using standard RT-PCR, our results show that DAT and SERT mRNAs are expressed and can be detected in adult zebrafish brains. Moreover, for SERT, two isoforms of the transporter (SERTa and SERTb) could be detected; however, SERTb shows less expression than SERTa (Figure S1). These results confirm that similar structures for dopamine and serotonin transporters are present in the fish and probably have a similar function. We also detect gene expression of GAPDH and ACTb1 as markers of neuronal activation.

Figure S1: PCR gel of GAPDH, DAT, SERTa, SERTb, and ACTb1.

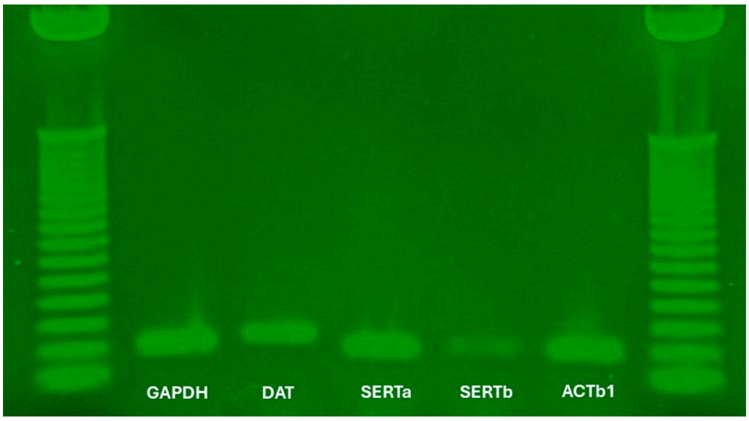

Fig. S1. In the zebrafish brain, mRNA expression levels of monoamine transporters (DAT, SERTa, SERTb) and positive controls of neuronal expression and activity, GAPDH and ACTb1.

To complement behavioral findings, conventional RT-PCR was conducted to verify the presence of monoaminergic transporter genes in the zebrafish CNS. We confirmed the expression of the dopamine transporter (DAT) and two serotonin transporter isoforms (SERTa and SERTb). Notably, SERTb exhibited lower expression levels relative to SERTa, suggesting that SERTa may represent the predominant isoform in the zebrafish CNS. These results support the utility of zebrafish as a model system for pharmacological and behavioral profiling of SERT and DAT-targeting compounds such as fluoxetine and methylphenidate. Additionally, expression of GAPDH, and ACTB1, implicated in intracellular signaling, was detected. The expression patterns of DAT, SERT, GAPDH, and ACTB1 in zebrafish were consistent with those observed in mammalian systems, further validating the translational relevance of this model.

Reverse Transcription and Polymerase Chain Reaction (RT-qPCR)

A total of 10 fish were separated into two groups. The fish were euthanized in ice-cold water and then dissected on ice to obtain their brains for analysis. Brains were pooled and homogenized to extract total RNA using the commercial SV Total RNA Isolation System kit (Promega, Madison, WI, USA) according to the manufacturer's instructions. Genomic DNA was removed from the samples using the commercial RQ1 RNase-Free DNase kit from Promega (Madison, WI, USA). The quality of the RNA was determined using a Varioskan Flash Multimode Reader from ThermoFisher Scientific (Waltham, MA, USA), which measured the 260/280 and 260/230 absorbance ratios. cDNA synthesis

was done using ImProm-II™ Reverse Transcription System from Promega (Madison, WI, USA). The genes actb1 and gapdh were selected as neuronal markers. The monoamine transporter genes evaluated were dat, serta, and sertb, and the markers were actb1 and gapdh. Primers of the genes used were designed by retrieving the sequences from the ZFIN database and using the web tool Primer BLAST to generate the primers (Table S1). To select the appropriate primers, the thermodynamic parameters were evaluated using the Oligo Analyzer tool from IDT DNA Technologies.

Table S1. Sequence of primers used in the RT-PCR analysis.

Primer	Bp	Sequences	
		Forward	Reverse
dat	116	GCCACCTTCAATCCTCCCAA	TTGTAGAAGGCGTAGAGCGG
serta	92	CAAAGCCCCAAAGAAGGTGC	TCAAGGCTCGTCTGTTGGAC
sertb	95	AGGAGACCAGCGTATGGGTA	GGGATTGTAGCTGGACAGGG
actb1	96	TGGGCGTCCATGACCTTTTT	ACTGGGCCAAGTTTAAGGCT
gapdh	103	CCGTCTTGAGAAACCTGCCA	AACCTGGTGCTCCGTGTATC
